# Voice rest and sick leave after phonosurgical procedures: surveys among European laryngologists and phoniatricians

**DOI:** 10.1007/s00405-019-05283-1

**Published:** 2019-01-10

**Authors:** Heikki Rihkanen, Ahmed Geneid

**Affiliations:** Department of Otorhinolaryngology and Phoniatrics, Helsinki University Hospital, University of Helsinki, Post Box 263, 00029 Helsinki, Finland

**Keywords:** Phonosurgery, Vocal fold surgery, Absolute voice rest, Relative voice rest, Sick leave

## Abstract

**Purpose:**

After surgery of vocal folds, almost every patient will need some voice rest. It is common to recommend total silence for some days, followed by less restricted voice use for variable periods. By now, we do not know how voice rest affects the healing process or the current practise in Europe.

**Methods:**

Members of the European Laryngological Society (2012) and the Union of European Phoniatrics (2018) were sent a web-based questionnaire which included two patient cases with a short history and a still picture. The respondents were asked about the postoperative recommendation of absolute voice rest and sick leave.

**Results:**

Over 90% of the respondents would recommend absolute voice rest after removing a polyp or after mucosal repair of Reinke’s oedema. For both cases, the mean length of recommended absolute voice rest among UEP members was 4 days (range 0–10 days) and among ELS members was 5 days (range 0–14 days). The recommended sick leave ranged from 0 to 35 days. The mean figures suggested by ELS members for the receptionist with Reinke’s oedema were 12 days and for the teacher with a polyp 13 days. On average, UEP members recommended 14 days of sick leave for both cases.

**Conclusion:**

The present scientific evidence is scant, but does not support for prolonged (over 3 days) absolute voice rest after simple phonosurgery. So far, there are no studies that could show absolute voice rest to be superior over relative voice rest. According to the present survey, there is considerable variation in recommending voice rest and sick leave after the removal of benign mucosal lesions. Many European laryngologists suggest voice rest that is longer and stricter than the present scientific literature supports.

## Introduction

After removing a benign vocal fold lesion, most patients will have a slightly hoarse voice due to the imperfect mucosal wave. Most feel vocal fatigue and some are unable to control the pitch and power for some days. Consequently, patients are advised to rest their voice and granted a sickness certificate.

Absolute voice rest implies that the patients are instructed to not use their voice either for speaking or whispering, and also to minimise any voice-use activities such as coughing or throat-clearing. Relative voice rest is less well defined [1]. It is common that, after vocal fold surgery, voice surgeons recommend either absolute or relative voice rest or both. In a survey among active US members of the American Academy of Otolaryngology-Head and Neck Surgery, half of the respondents recommended absolute voice rest after the removal of benign vocal fold lesions, 15% did not favour any type of voice rest [2].

Koufman and Blalock [3] published a retrospective case series with 93 patients who had been recommended relative voice rest (voice conservation technique) and 33 patients with absolute voice rest. The study shows that preoperative voice counselling correlated with a better outcome, but absolute voice rest provided no greater protection against postoperative dysphonia than relative voice rest. In a recent, randomized study [4] phonosurgical patients were divided into two groups. The first group (*N* = 16) was recommended for 3-day and the second (*N* = 15) for 7-day absolute voice rest. Both groups received voice therapy after the silent period. Jitter, shimmer, and VHI-10 were significantly better in the 3-day group at 1 month after operation. In addition, GRBAS grading was significantly better in the 3-day group at 1 and 3 months. Moreover, in the stroboscopic examination, normalised mucosal wave amplitude was significantly more often seen in the 3-day group at 1, 3, and 6 months post-operation compared with the 7-day group. The result favours short voice rest.

The review by Ishikawa and Thibeault [5] summons the biology of vocal fold wound healing. In rats and rabbits, the inflammatory process starts readily and the peak of fibroblast proliferation takes place on the third day. It is not known how the vocal fold mucosa should be mechanically strained at that point. However, if we follow the reasoning from the other fields of medicine and the healing process of a vocal fold, we could agree that overloading and excessive mechanical stress to the mucosa are not beneficial [5]. We do not know whether light exercise (relative voice rest) is better than no voicing at all (absolute voice rest). We should, however, be aware that patients do not necessarily follow all our recommendations. According to the voicing dosimeters, patients reduce their voice loading, but are not silent [6]. For time being, there is not a consensus on the type and length of voice rest after vocal fold surgery.

Most people need their voice and speech at their work. After phonosurgery, the length of sick leave (days out of work) depends on several factors. It is related to the profession, working place, but also to the national healthcare and reimbursement policies. Sick listing practises are often learnt during the specialisation period, without structured education, and are known to vary [7]. Assessments of the ability to return back to work after phonosurgery are missing. It is important to realise that the cost of sick leave might overweight all the other expenses caused by the vocal fold surgery. Therefore, it is important to get an idea of the magnitude of variability in postoperative recommendations among laryngologists and phoniatricians.

## Materials and methods

**Fig. 1:**
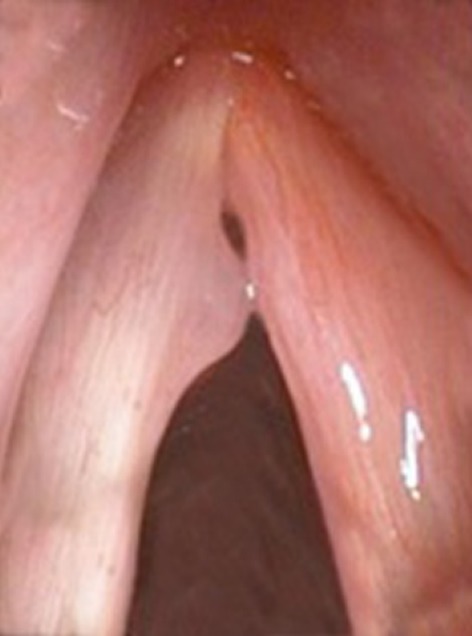
28-year-old female teacher. Hoarse and rough voice for 6 months. In indirect/direct office laryngoscopy, you see a vocal fold polyp

**Fig. 2:**
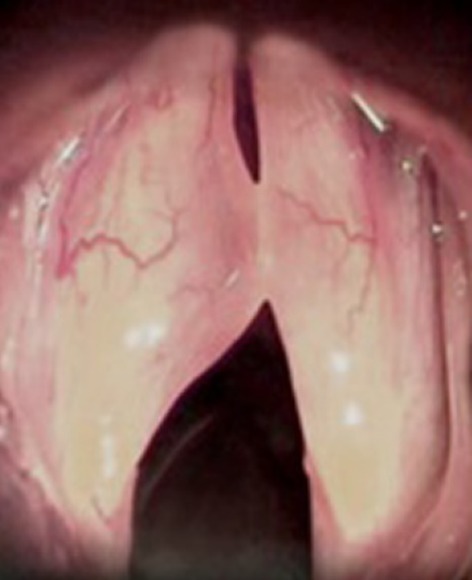
62-year-old female receptionist. Heavy smoker, 1 pack/day for 35 years. Hoarse and masculine voice. In indirect/direct office laryngoscopy, you see Gradus 3 Reinke’s oedema. She has stopped smoking 1 month ago

The members of the Union of European Phoniatricians (UEP) were sent a web-based questionnaire in spring 2018. They were shown a still photo of a benign vocal fold lesion with a short patient history (Figs. 1, 2). The respondents were asked how you would help the patient. In case you choose to operate, for how many days you suggest absolute voice rest (options 0–14 days) and for how long you recommend to stay out of work (options 0 days—more than 5 weeks). The days of absolute voice rest and sick leave were analysed. Answers were multiple choice options. The response rate after two reminders was 23%. These results are briefly compared to an earlier questionnaire sent to the members of the European Laryngological Society (ELS) in spring 2012. The cases were identical, but the length of absolute voice rest and sick leave were open for any number. The response rate after two reminders of the ELS questionnaire was 29%. Since some of the doctors are members of both societies, it is likely that a few responses in 2018 were given by the same person as in 2012.

## Statistical analysis

Statistical analyses were performed using SPSS version 22 (Chicago, IL, USA). Spearman’s rho was applied to find a correlation between the suggested voice rest and the sick leave or a correlation between the recommendations for the two presented cases. A 95% confidence level was used to indicate the statistical significance.

## Results

Members of the UEP choose to remove the teacher’s polyp in 89% of the answers and to treat the receptionist’s Reinke’s oedema surgically in 88% of the responses.

**Fig. 3:**
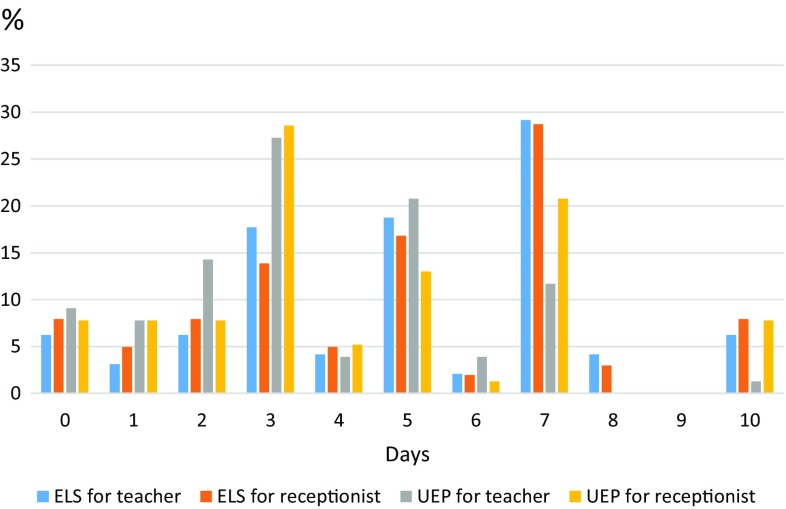
Recommended days of absolute voice rest after the procedure. Figures are presented as proportion of the each colour group. There were two ELS members (2%) who recommended 14 days of absolute voice rest. This is omitted from the graph

Over 90% of the respondents would recommend absolute voice rest after removing the polyp or mucosal repair of the Reinke’s oedema. On average, the length of absolute voice rest among UEP members was 4 days and among ELS members 5 days for both cases. The range among UEP members was 0–10 days, and among ELS members 0–14 days. The variation is large, as shown in Fig. 3. 8% of UEP members and 7% of ELS members did not find absolute voice rest necessary.

**Fig. 4:**
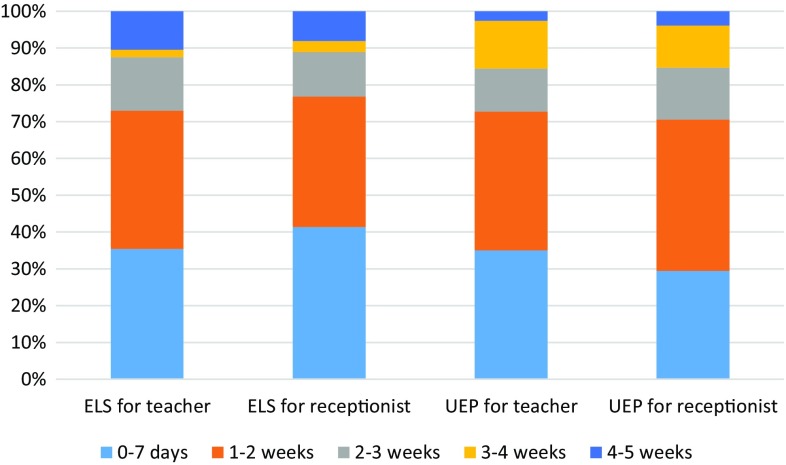
Recommended sick leave after the procedure. Figures are presented as proportion of the answers within each group

The mean length of sick leave for the teacher was 13 days and for the receptionist 12 days among the respondents of the ELS. UEP members recommended sick leave, on average, for 14 days in both cases. The range among UEP members was 1–35 days and among ELS members 0–30 days (Fig. 4).

**Fig. 5:**
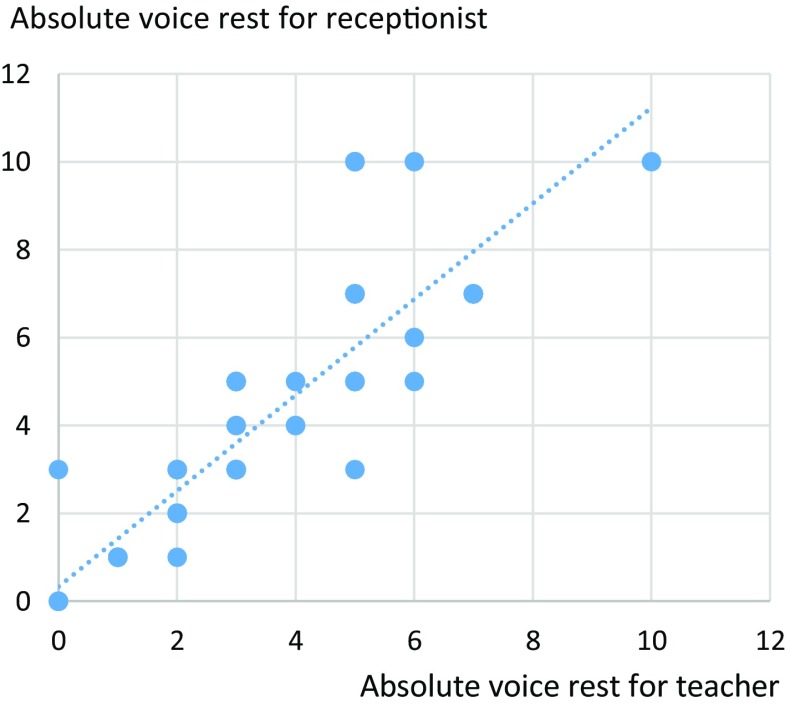
Days of recommended absolute voice rest for the teacher after removing the polyp and for the receptionist after surgical treatment of Reinke’s oedema, as suggested by UEP members

We found no correlation between the length of absolute voice rest and length of sick leave recommended by UEP members. However, the UEP members were quite consistent in recommending absolute voice rest both for the receptionist and for the teacher (Spearman’s rho 0.915, *p* < 0.001) (Fig. 5), as well as the length of sick leave (Spearman’s rho 0.866, *p* < 0.001).

## Discussion

Absolute voice rest is a burden to the patient [8], and we know that most patients do not follow the recommendation [6, 8]. There has been increasing criticism for the strict restriction of vocalisation after phonosurgical procedures. According to a recent, randomised study, both short- and long-term vocal recoveries were similar regardless of the recommended 7-day absolute or relative voice rest [6]. One randomised study showed that extending absolute voice rest over 3 days declines the outcome [4]. One randomised study [9] showed that the group with 10 days’ absolute voice rest (*N* = 16) had similar voice results as the group with 5 days (*N* = 15), except in the improvement of maximum phonation time (MPT). That was better in the group of prolonged absolute voice rest. There are, however, no studies that would show absolute voice rest to be superior over relative voice rest after simple phonosurgical procedures. Postoperative recommendations after more sophisticated laryngeal procedures (e.g., sutured of mucosal flaps or grafts) have not been studied. It is presumed that different surgical approaches will need different kinds of postoperative care. Poor patient compliance makes it difficult to assess the benefit of specific recommendations, especially the effect of absolute voice rest after phonosurgery.

In 2010, the thorough review by Ishikawa and Thibeault [5] states that the current literature lacks clinical evidence to support a specific type and duration of voice rest. Even today, more studies are needed. According to the website of Clinical Trials (https://clinicaltrials.gov/), there are three registered, controlled studies recruiting patients for studies on voice rest after phonosurgery. In one of these studies, all study arms contain absolute voice rest; two compare absolute voice rest with voice use.

The response rates in the present studies are low (23% and 29%), but slightly greater than obtained in a questionnaire sent to the US otorhinolaryngologists (16.9%) [2]. The result might not represent the general conception of the field, but, nevertheless, show the great variability of postoperative recommendations. The vast majority of respondents of both European specialist organisations advocate absolute voice rest after simple phonosurgery. Absolute voice rest extending over 3 days was recommended in 42% of the UEP-survey responses. By now, there are no scientific data to support this. The present knowledge and information on patient compliance would advocate either short absolute restriction of voicing or relative voice rest only. The latter means that the person should speak softly, avoid long voicing, and restrict speaking in noisy environment, on phone, or outdoors to the minimum.

We found no correlation between the recommended absolute voice rest and sick leave. This suggests that the respondents considered these two as independent factors, not a continuum of the healing process.

All phonosurgical patients will need some kind of voice rest. This prevents working in many professions. Thus, most patients will need a certificate for sickness. In the present study, the respondent was given a still picture and a very brief but uniform patient history. The large variation in the length of sick leave signifies different traditions, beliefs, and cultures in the medical rehabilitation. Since we have no information of the respondents’ nationality, further analysis of regional or national differences is not possible.

The total expense of removing a benign vocal fold lesion will, in any case, be doubled or tripled by the cost of the sick leave. There are several ways to calculate the cost of a lost working day. In Finland, the cost of a short sick leave to the employer in 2012 was calculated to have been 193 €/day. This was done by the government officials (https://stm.fi/documents/1271139/1332445) knowing the total number of missed work days and the real salaries of the sick. With an average salary, the total cost has now been estimated to exceed 400 €/day by Finnish employees’ insurance companies Etera and Varma (https://www.varma.fi/muut/laskurit/poissaololaskuri/). Thus, a certificate within the range of one standard deviation (14 days ± 8 days) of our study would mean that the cost of sick leave would vary from 2000 to 8400 €, in case the patient had been Finnish. To be fair to our patients and their employers and to justify our decisions, we need more studies and guidelines to unify our traditions in granting sick leave after phonosurgical procedures.

## Conclusion

By now, there is no scientific evidence to show that absolute voice rest is superior over relative voice rest after simple phonosurgery. If absolute voice rest is recommended, the scant medical literature favours a short rest (3 days) rather than extending it over several days. This study shows that European voice surgeons recommend absolute voice rest very frequently and extend it over 3 days. Accordingly, most laryngologists and phoniatricians in Europe should re-evaluate their patient guidance after simple ablation of vocal fold mucosal lesions. However, more randomised studies are needed before guidelines for voice rest and sick leave after vocal fold surgery can be established.
